# SARS-CoV-2 Antibody Profiles in Maternal Serum and Breast Milk Following mRNA COVID-19 Vaccination: A Longitudinal Prospective Observational Cohort Study

**DOI:** 10.3390/vaccines11111643

**Published:** 2023-10-26

**Authors:** Hui-Mien Hsiao, Langdon S. DiMaggio, Maria A. Perez, Xuemin Chen, Kathleen Stephens, Theda Gibson, Evan J. Anderson, Christina A. Rostad

**Affiliations:** 1 Department of Pediatrics, Emory University School of Medicine, Atlanta, GA 30322, USA; hhsiao@emory.edu (H.-M.H.); langdon.smythe.dimaggio@emory.edu (L.S.D.); maria.de.los.angeles.perez.lizasuain@emory.edu (M.A.P.); xchen26@emory.edu (X.C.); kasteph@emory.edu (K.S.); theda.gibson@emory.edu (T.G.);; 2 Center for Childhood Infections and Vaccines, Children’s Healthcare of Atlanta, Atlanta, GA 30322, USA

**Keywords:** pregnancy, lactation, fetal, neonatal, IgG, IgA, pseudovirus, neutralization, kinetics, variant

## Abstract

COVID-19 vaccination during pregnancy protects infants against symptomatic COVID-19. Vaccination of lactating mothers may offer additional protection, but our understanding of immune responses in breast milk is limited. We, therefore, performed a single-center prospective cohort study of lactating mothers who received a COVID-19 mRNA primary vaccine series to evaluate the durability, breadth, and neutralizing capacity of the antibody responses in breast milk. Spike IgG- and IgA-binding antibodies of ancestral SARS-CoV-2 in serum and breast milk were quantified over 9 months using Meso Scale Discovery (MSD) V-PLEX assays, and ancestral titers were compared to four variants of concern (Alpha, Beta, Delta, Gamma) at a single time point. Neutralizing antibodies against ancestral SARS-CoV-2 and Omicron BA.4/5 were compared before and after vaccination using a pseudovirus-neutralization assay. Eleven lactating mothers received either Pfizer BNT162b2 (7/11) or Moderna mRNA-1273 (4/11) vaccine primary series. IgG and IgA titers increased in serum and breast milk following each dose, peaking 1–4 weeks after series completion. Titers remained significantly elevated for 7–9 months, except for in breast milk IgA which returned to baseline within 1 month. Furthermore, binding antibodies against all included variants were detected in breast milk collected 1–3 weeks after series completion. However, while vaccination induced a strong neutralizing response against ancestral SARS-CoV-2 in serum and more modest response in breast milk, it did not induce neutralizing antibodies against Omicron BA.4/5 in either specimen type. This study demonstrates that maternal COVID-19 mRNA vaccination may enhance immune protection for infants through breast milk via increased IgG- and IgA-binding-and-neutralizing antibodies; although, variant-specific boosters may be required to optimize immune protection.

## 1. Introduction

Since severe acute respiratory syndrome coronavirus 2 (SARS-CoV-2) emerged in 2019, leading to hundreds of millions of infections and millions of deaths worldwide, multiple vaccines have been developed to curb the morbidity and mortality of coronavirus disease 2019 (COVID-19). Vaccinations were estimated to have prevented 20 million deaths from COVID-19 from December 2020 to December 2021 [1]. Since then, further progress has been made, including the authorized emergency use of vaccinations for US children as young as 6 months of age in June 2022.

Unfortunately, infants less than 6 months of age are completely unvaccinated, and vaccination uptake in older infants and young children remains substantially lower than in their adult counterparts. As of May 2023, only 13% of US children aged 6 months to 4 years had received at least one dose of a COVID-19 vaccine [2], whereas approximately 70% of the total US population had completed a primary series [3]. Furthermore, infants have an immature immune system that takes months to develop and years to fully mature. Throughout the pandemic, infants less than 6 months of age have been hospitalized with COVID-19 at higher rates than children 6 months to 4 years old, and when Omicron surged, infants under 6 months old accounted for 44% of pediatric hospitalizations [4].

Therefore, it is essential to identify strategies to protect this vulnerable, unvaccinated population from COVID-19. Maternal vaccination likely protects infants via transplacental antibody transfer and reduced transmission of infection from mother to infant. Immunity provided by the breast milk of vaccinated mothers could further provide a valuable means of passive immunity for infants. This approach has been evaluated for other respiratory pathogens, including pertussis and influenza, for which secretory IgA and IgG in breast milk has been found to protect infants from these respective respiratory diseases [5]. Though antibody detection in breast milk of mothers following COVID-19 infection or vaccination has been reported previously, limited data is available regarding their longevity, functional capacity, or response to emerging variants. This study, therefore, aimed to elucidate the magnitude, kinetics, and breadth of SARS-CoV-2 antibodies in breast milk from COVID-19-vaccinated mothers.

## 2. Materials and Methods

### 2.1. Participant Enrollment and Sample Collection and Processing

Lactating mothers between 25 and 45 years of age who intended to receive a COVID-19 mRNA vaccine in Atlanta, Georgia, USA, were eligible to enroll in this prospective observational cohort study. All participants provided written informed consent, and the study was conducted with approval from the Emory University Institutional Review Board (IRB). Participants received either the Moderna mRNA-1273 or Pfizer BNT162b2 mRNA vaccine primary series. Participants were not excluded based on prior COVID-19 diagnosis or exposure. Breast milk and serum samples, described below, were collected from January 2021 to January 2022. Samples were ideally collected prior to vaccination, between the first and second dose, 1–3 weeks after the second dose (“Post 2”), and at 1, 2, 3, 4–6 months, and 7–9 months after the second dose. On occasion, participants provided multiple breast-milk samples within a particular time point, in which case only the later sample was used. Serum samples were analyzed 3–4 weeks after dose one, while breast milk samples were analyzed at both 1–2 weeks and 3–4 weeks after dose one (Supplemental Data, Table S1). All samples were collected prior to receipt of any booster vaccination doses.

Participants provided fresh or frozen breast-milk samples, which were collected independently using their preferred method into sterile containers. Serum samples were collected in serum-separator tubes and processed with standard phlebotomy methods. Prior to storing breast milk, the aqueous layer was separated from the fat layer with centrifugation at 800× *g* at 4 °C for 10 min, as previously described [6,7]. The aqueous layer of breast milk and the serum samples were aliquoted and stored at −80 °C until analysis.

### 2.2. Meso Scale Discovery (MSD) Assays to Detect IgG and IgA Levels in Serum and Breast Milk

V-PLEX SARS-CoV-2 Panel 13 or Panel 24 IgG and IgA 96-well Plate Serology Kits (Meso Scale Diagnostics, LLC; Rockville, MD, USA, K15463U, K15465U, K15575U) were used to measure binding-IgG and -IgA antibodies to SARS-CoV-2-spike variants, including the ancestral strain (Wuhan-hu-1), Alpha (B.1.1.7), Beta (B.1.351), Delta (B.1.617.2), and Gamma (P.1), in heat-inactivated serum and breast-milk samples. Assays were performed according to manufacturer protocol; although, the assay has not been fully evaluated for breast milk. In brief, plates were processed and incubated with serially diluted serum or breast-milk samples then with an electrochemiluminscent label (SULFO-TAG anti-human IgG or IgA antibody) and analyzed on the MESO QuickPlex SQ 120MM (Meso Scale Diagnostics, LLC; Rockville, MD, USA). Titers were calculated against a reference standard calibration curve and reported as arbitrary units (AU)/mL.

### 2.3. Production of Pseudovirus and Titration Assay

To construct SARS-CoV-2 pseudoviruses, a recombinant plasmid containing the spike protein gene from ancestral (Wuhan-hu-1) SARS-CoV-2 or Omicron BA.4/5 was co-transfected with an HIV-1 lentiviral backbone plasmid (pNL4.3.Luc.R-E-) into 293T cells, and then harvested from the supernatant, as previously described [8]. Pseudoviruses were filtered (0.45-µm pore size) and stored at −80 °C until use. Pseudoviruses were titrated using a luminescence read-out on a TopCount^®^ NXT^TM^ luminometer (Packard Instrument Company; Meriden, CT, USA).

### 2.4. Ancestral SARS-CoV-2 and Omicron BA.4/5 Pseudovirus-Based Neutralization Assay

Pseudovirus-neutralization assays were performed as previously described [8,9]. 293T-ACE2 cells were cultured in Dulbecco’s Modified Eagle Medium (DMEM) (Gibco^TM^ 11995065) with 10% fetal bovine serum (FBS) (Bio-techne^®^ S11150H) and 200 µg/mL of hygromycin then seeded in 96-well plates (2 × 10^4^ cells/well) in 100 µL volume and incubated at 37 °C overnight. The following day, heat-inactivated serum samples were diluted in 3-fold serial dilution starting at 1:30 in 5% FBS DMEM, and heat-inactivated breast milk samples were diluted in 3-fold serial dilution starting at 1:10 in 5% FBS DMEM. An equal volume of approximately 3 × 10^4^ RLU/mL SARS-CoV-2 pseudoviral particles were added, and the sample-virus plates were incubated at 37 °C for 1 h. Media were removed from the plates before 100 µL of this sample-virus mixture was added to the cells. Uninfected cells and plain SARS-CoV-2 pseudovirus were used as cell-controls and virus-controls, respectively, on each plate. Plates were incubated for 48 h at 37 °C with 5% CO_2_. Then, 100 µL of Britelite™ Plus luciferase substrate (Perkin Elmer^®^ 6066769) was added per well and relative luminescence units (RLUs) were analyzed on a TopCount^®^ NXT^TM^ luminometer (Packard Instrument Company; Meriden, CT, USA). Pseudovirus-neutralization curves were generated using 4-point non-linear regression analysis, and titers were expressed as the effective serum or breast milk concentration at which 50% of the virus was neutralized (EC50). The lower limit of detection (LLOD) was defined as the starting dilution of 1:30 or 1:10. Undetectable titers were assigned a value of ½ LLOD (1:15 or 1:5).

### 2.5. Statistical Analyses

Statistical analysis was performed in GraphPad Prism version 9.5.1. Log-transformed antibody titers were compared using a mixed-effects model or one-way ANOVA with Friedman post hoc comparisons for binding-antibody analyses. For neutralization analysis, EC50s were compared using a Mann–Whitney test of log-transformed values. Linear regression analysis of the log-transformed antibody titers was performed for IgG and IgA in serum and breast milk to determine, R^2^, the coefficient of determination. *p*-values ≤ 0.05 were considered statistically significant.

## 3. Results

### 3.1. Clinical Cohort Characteristics

Eleven lactating mothers intending to receive either the Pfizer BNT162b2 vaccine or Moderna mRNA-1273 vaccine primary series were enrolled into this cohort study (Table 1). The mean age of mothers was 33 years old (range 27 to 42 years old). Two identified as being of black or black/white race and nine identified as being of white race. Samples were collected from 15 January 2021 to 7 January 2022, during which time the Delta variants became predominant, and was largely prior to the Omicron variant emergence in December 2021. Overall, 59 serum samples and 65 breast-milk samples were collected from the 11 participants for analysis. Seven participants received the Pfizer mRNA vaccine and four received the Moderna mRNA vaccine primary 2-dose series. Most participants (8/11) were vaccinated after giving birth, and their infants were 2 weeks to 3 months of age at the time of the mother’s initial vaccination. Three participants were vaccinated while pregnant, from 1 week to 2 months before delivery. Participants were not excluded based on prior COVID-19 illness or exposure. One participant was known to have been exposed to COVID-19 prior to participation. Due to the nature of this study and sample collection during the COVID-19 pandemic, samples from each time point were not available for all participants. Participants were included regardless of the number of samples available.

### 3.2. SARS-CoV-2-Binding Antibodies in Serum and Breast Milk

**Figure 1:**
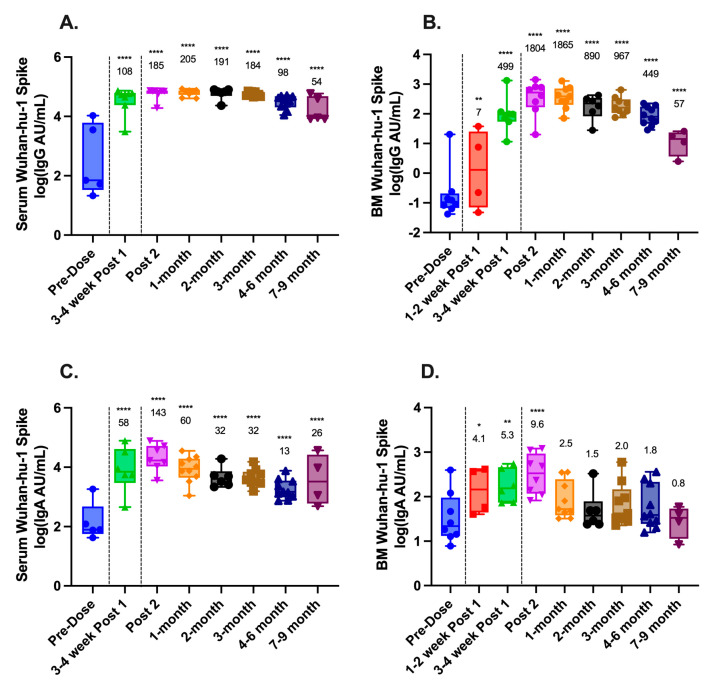
Longitudinal antibody responses to SARS-CoV-2 in maternal serum and breast milk following COVID-19 vaccination. IgG and IgA titers to ancestral SARS-CoV-2 (Wuhan-hu-1) spike proteins were measured using Meso Scale Discovery (MSD) V-PLEX assays and reported as arbitrary units (AU)/mL. (**A**) Serum IgG; (**B**) breast milk (BM) IgG; (**C**) serum IgA; and (**D**) breast milk (BM) IgA. Black dashed lines represent vaccine administration. Geometric mean fold rise (GMFR) compared to pre-vaccine baseline is shown above each time point. Statistical comparisons were calculated using a mixed-effects model of log-transformed values. Asterisks represent the statistical comparison of each group with the pre-vaccine titer, * *p* < 0.05; ** *p* < 0.01; **** *p* < 0.0001.

MSD assays were used to determine concentrations of IgG and IgA antibodies to ancestral SARS-CoV-2 (Wuhan-hu-1) spike antigens in the serum and breast milk of lactating mothers before and after vaccination. As expected, IgG and IgA titers in both serum and breast milk significantly increased following vaccination (Figure 1A–D). Compared to pre-vaccine baseline titers, serum IgG titers had a geometric mean fold-rise (GMFR) of 108-fold following dose one and 185-fold following dose two, with a peak of 205-fold at 1-month post dose two. Similarly, serum IgA titers had a GMFR of 58-fold following dose one and peaked at 143-fold following dose two. In breast milk, IgG antibodies had a GMFR 499-fold following dose one and peaked with an 1865-fold increase at 1-month post dose two. In contrast, breast milk IgA had a GMFR which, compared to baseline, was modest, rising only 5.3-fold after dose one and peaking with a 10-fold increase following dose two.

In both serum and breast milk, IgG levels remained statistically elevated compared to the baseline at 7–9 months post dose two (*p* < 0.0001 for all comparisons) (Figure 1A,B). Serum IgA also remained statistically elevated compared to the baseline at 7–9 months (*p* < 0.0001) (Figure 1C), whereas breast milk IgA returned to baseline levels within 1 month of vaccination (Figure 1D).

### 3.3. SARS-CoV-2 Pseudovirus-Neutralizing Antibodies in Serum and Breast Milk

**Figure 2:**
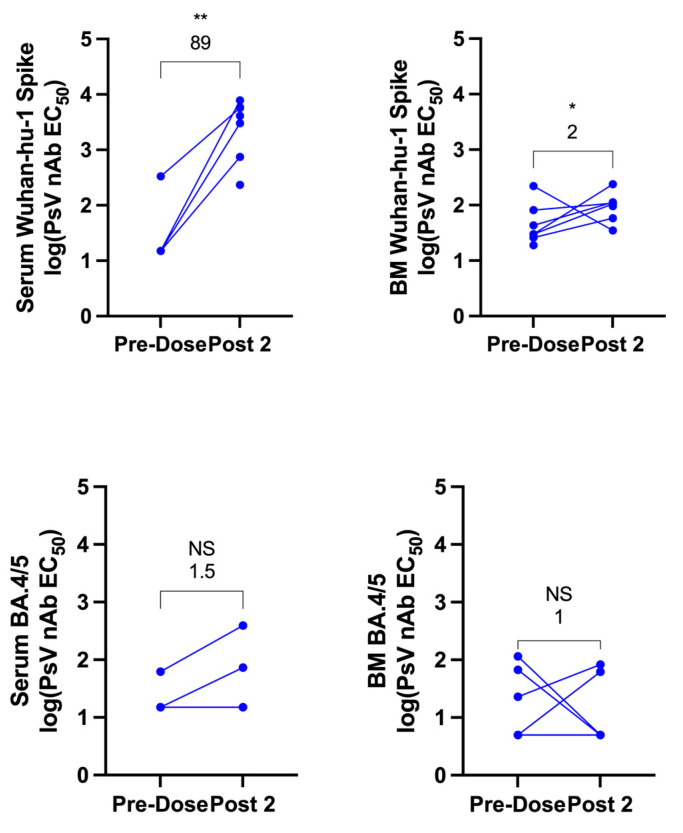
Ancestral and Omicron BA.4/5 SARS-CoV-2-pseudovirus neutralization in maternal serum and breast milk before and after COVID-19 vaccination. Pseudovirus-neutralizing antibody (PsV nAb) titers to ancestral SARS-CoV-2 (Wuhan-hu-1) or Omicron BA.4/5 spike protein prior to vaccination and following completion of the primary vaccine series (1–3 weeks post dose two) were generated for serum and breast milk (BM) and reported as estimated concentration at which 50% of pseudoviruses were neutralized (EC50). The GMFR compared to pre-vaccine baseline is shown above each graph. Statistical comparisons were calculated using a Mann–Whitney test of log-transformed values. Asterisks represent the statistical comparison of each group with the pre-vaccine titer, * *p* < 0.05; ** *p* < 0.01, NS not significant. Note, an equal number of breast milk and serum samples were analyzed for Omicron BA.4/5 but three of these are overlapping for serum as levels were undetectable both before and after vaccination.

Next, pseudovirus neutralization was analyzed to determine the neutralizing capacity of antibodies in serum and breast milk against ancestral SARS-CoV-2 pseudovirus and against Omicron BA.4/5. The pseudovirus-neutralizing antibody EC50 was compared between pre-vaccine and post-vaccine (Post 2) samples in both serum and breast milk. Samples were included for each time point if available, regardless of whether a matched sample was available from the other time point. One breast milk sample was excluded due to evidence of bacterial contamination following incubation at 37 °C. In serum, neutralizing-antibody responses against ancestral SARS-CoV-2 increased with a GMFR of 89 above baseline (*p* < 0.01) after vaccination. Breast-milk neutralizing-antibody response increased as well, though by only 2-fold (*p* < 0.05) (Figure 2). Neutralizing-antibody response to Omicron BA.4/5 following vaccination was not significant in serum or breast milk.

### 3.4. SARS-CoV-2-Variant Antibodies in Serum and Breast Milk

**Figure 3:**
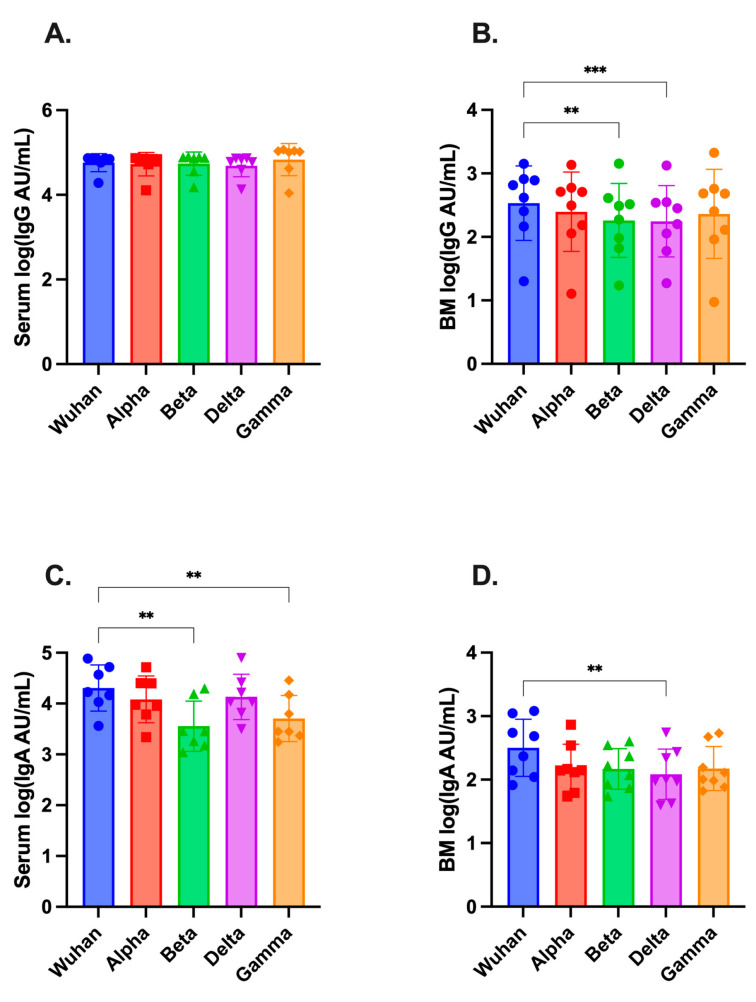
Antibody titers against SARS-CoV-2 variants in maternal serum and breast milk following COVID-19 vaccination. MSD assays were used to measure binding antibody titers to Alpha, Beta, Gamma, and Delta SARS-CoV-2 variants in (**A**) serum and (**B**) breast milk (BM) for IgG; and (**C**) serum and (**D**) breast milk for IgA and reported as arbitrary units (AU)/mL. Samples were analyzed 1–3 weeks post dose two, when antibody responses were at or near their peak. Statistical comparisons of log-transformed titers were performed using one-way ANOVA with Friedman post hoc analysis. Asterisks represent the statistical comparison of Wuhan-hu-1 against variant, ** *p <* 0.01; *** *p* < 0.005.

MSD assays were further used to compare ancestral SARS-CoV-2 binding IgG- and IgA-antibody titers to those of circulating variant spikes, specifically Alpha (B.1.1.7), Beta (B.1.351), Delta (B.1.617.2), and Gamma (P.1). This analysis was also performed at the post-dose-two time point (Figure 3A–D). Both IgG and IgA were found in all four variants in serum and breast milk. Serum IgG-variant antibody titers were similar to those of the ancestral strain (Figure 3A). Breast milk IgG exhibited statistically significant decreases in titer for Beta (*p* < 0.01) and Delta (*p* < 0.001) (Figure 3B). Serum IgA was significantly lower for Beta and Gamma variants compared to Wuhan-hu-1, while breast milk IgA was lower for Delta (*p* < 0.01 for all comparisons, Figure 3C,D).

### 3.5. Correlation between SARS-CoV-2 Serum and Breast-Milk Antibodies

**Figure 4:**
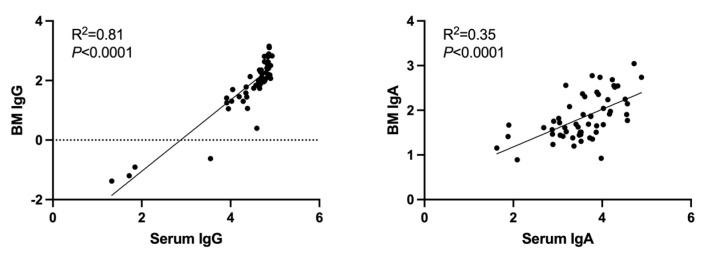
Correlation between ancestral SARS-CoV-2-spike protein antibody titers in maternal serum and breast-milk samples. Linear regression analysis of the log-transformed antibody titers was performed for IgG and IgA in breast milk (BM) and serum. R^2^ is the coefficient of determination.

Finally, linear regression analysis of the log-transformed antibody titers was performed for IgG- and IgA-neutralizing antibodies to compare serum and breast-milk samples (Figure 4). The coefficient of determination (R^2^) was 0.81 for IgG in breast milk versus serum and 0.35 for IgA in breast milk versus serum (*p* < 0.001 for both comparisons). This indicates a strong correlation between IgG levels in breast milk and serum, and a lesser but still significant correlation in IgA levels.

## 4. Discussion

Our study demonstrates that lactating mothers who received a primary series of either Moderna or Pfizer COVID-19 mRNA vaccines exhibited significant IgG and IgA responses not only in their serum, but in their breast milk as well. This is corroborated by previous studies that have shown IgG and IgA responses to SARS-CoV-2 infection [10,11,12] and to mRNA vaccination in both the serum and breast milk of lactating mothers [6,10,12,13,14,15,16,17,18,19,20,21]. It has also been noted that while natural infection produces a robust IgA response [11,12,13,21,22,23], vaccination produces an IgG-dominant response with a lesser IgA response [11,12,13,19,21]. This is not unexpected, given that infection is mediated by mucosal exposure, eliciting an IgA response, whereas vaccination is administered intramuscularly, inducing a more predominant IgG response.

We next demonstrated the kinetics of IgG and IgA concentrations in breast milk over time. In our study, IgA titers in both serum and breast milk peaked within the first 3 weeks of completing the primary vaccine series, while IgG peaked at 1 month. This timing is generally supported by the literature; although, IgG in breast milk has been previously shown to peak earlier, closer to 1–3 weeks after the second dose. This is likely explained by the inter-individual variability noted in our study and those cited and by the relatively small difference between GMFR for IgG at the post 2- and 1-month time points. Linear regression analysis of the log-transformed antibody titers demonstrated a high correlation between IgG antibodies in breast milk and serum (R^2^ = 0.84) and a moderate correlation for IgA antibodies (R^2^ = 0.35).

Furthermore, while the majority of prior studies ended within 6 weeks of the second vaccine [6,18,21,24], we demonstrated that IgG titers remained elevated in breast milk throughout the course of the 7–9-month follow-up, correlating with serum titers. In contrast, IgA titers in breast milk declined more quickly, returning to baseline levels within just one month of completing the primary vaccine series. These results are consistent with previous studies that continued for 2 to 5 months after the second dose [7,12,25] and suggest that vaccinated mothers provide passive SARS-CoV-2-specific antibodies to their infants through breast milk, with the greatest benefit observed early after vaccination. Furthermore, IgG antibodies in breast milk may provide protection for many months after vaccination. These data are in contrast to the study by Golan et al., which reported persistently elevated anti-SARS-CoV-2-spike antibody levels in human milk up to 8 months after vaccination, but found that IgG levels declined more notably than IgA levels [22]. 

The protective capacity of human breast milk against SARS-CoV-2 is further supported by the pseudovirus-neutralization analysis, which reveals an increase in neutralizing-antibody titers following vaccination, though by a notably lesser magnitude than serum. Pace et al. have previously reported microneutralization by breast milk in vitro following infection [11]. Several additional studies have demonstrated neutralization activity in breast milk following vaccination, employing pseudovirus, microneutralization, and surrogate neutralization assays. Collectively, they suggest that neutralizing antibodies rise later in breast milk than in serum but are significantly elevated 1 week after completing the primary vaccine series and that neutralizing antibodies persist for at least 3 weeks [12,14,19,21,26,27].

Since the initial outbreak of SARS-CoV-2, new variants of concern have emerged over time, including Alpha, Beta, Gamma, Delta, and Omicron. Alpha predominated in the first half of 2021, when sample collection began, and Delta predominated from summer 2021; until Omicron surged in late December 2021, shortly before sample collection was completed. Here, we demonstrated that the primary series of maternal mRNA vaccination elicited broad IgG and IgA antibodies that bound to all four variant strains included in this analysis (Alpha, Beta, Delta, Gamma) in both serum and breast milk. Low et al. similarly reported breast milk IgA antibodies against RBDs across these four variants of concern, though with significant reductions for Beta, Delta, and Gamma [28]. Though our study and others have noted significant variances in the response to some variants of concern, these results indicate that maternal vaccination with a primary mRNA series generally elicits cross-reactive antibodies to multiple SARS-CoV-2 variants that are secreted into the breast milk and passively transferred to the infant.

As Omicron and its subvariants have predominated through the current season, neutralization capacity was evaluated against BA.4/5, which was labeled a “variant of concern” in May 2022 [29], about 1 to 1.5 years after our participants’ first vaccine doses. Neither serum nor breast milk demonstrated a neutralizing response to Omicron BA.4/5. While other studies have described neutralizing activity against Alpha, Beta, and Gamma in breast milk after the primary vaccine series [14,19], our data contributes to a limited pool of information regarding the reduced neutralizing capacity of breast milk against Omicron following primary series vaccination. This includes a study by Olearo et al. which included only five vaccinated or vaccinated/recovered participants and demonstrated detectable neutralizing titers against Omicron B.1.529 but with a fourfold reduction compared to an early pandemic isolate [10]. Bäuerl et al. also reported that none of the breast milk samples of women infected in the Wuhan-hu-1 wave of COVID-19 and only 1 of 16 (6.25%) breast milk samples from women vaccinated with mRNA-1273 demonstrated neutralizing activity against Omicron BA.1 [26]. The lack of neutralization capacity to Omicron underscores the likely importance of variant-specific boosters and ongoing maternal vaccination. This is of particular importance now as pregnant and lactating women are offered the newly approved and available monovalent XBB.1.5-containing vaccines.

This study is limited by its small sample size and by the fact that samples could not be collected at all time points from all participants. This may magnify the effect of inter-individual variability, which has been noted previously [12,19,24,25]. The small sample size also prevented statistical comparisons between the two different vaccines. Future studies involving more participants could be informative. Another limitation is that the MSD assay used to measure binding antibodies has not been fully evaluated using breast milk. Furthermore, breast-milk-collection methods used by mothers were not standardized, which may have introduced variability in the quality of specimens obtained. For example, one participant’s breast milk was excluded from neutralization analysis due to suspected bacterial contamination after incubation at 37 °C. Finally, mothers were not excluded or stratified based on COVID-19 exposure or illness prior to or during the study. Therefore, natural infection could have been a confounder in this analysis.

Valuable future studies would include evaluation of samples following variant-specific booster doses of COVID-19 vaccines, which have now been authorized for use, and further exploration of cross-reactivity to the SARS-CoV-2 Omicron variant and its sublineages. New studies are also needed to determine how the immune properties in mothers’ breast milk translate to protection of breast-fed infants and children, including protection against newly emerging SARS-CoV-2 variants. In limited prior studies, anti-RBD antibodies have been detected in the stool, oral mucosa, and saliva of breastfeeding infants following maternal COVID-19 vaccination [19,21,22,30]. Expanding our knowledge of passive immunity to infants will better enable us to protect this vulnerable infant population.

While the American Academy of Pediatrics (AAP) and World Health Organization (WHO) recommend exclusive breast feeding for the first 6 months of life and support supplementary breast feeding until 2 years old, only 55.8% of infants are receiving any breast milk at 6 months of age and only 24.9% are exclusively breast fed [31]. Additionally, pregnant women were not included in initial vaccine clinical trials, and pregnant individuals have been less likely to receive vaccines compared to their non-pregnant counterparts [32], even though COVID-19 in pregnancy is associated with increases in maternal morbidity and mortality and neonatal complications [33]. Here, we expand upon the current understanding of human-breast-milk immunogenicity following COVID-19 vaccination of pregnant individuals, which may help shape prevention strategies against COVID-19 in this vulnerable population and inform the decisions pregnant individuals and mothers make regarding vaccination and breast feeding.

## 5. Conclusions

Maternal vaccination with mRNA COVID-19 vaccines induced broadly cross-reactive serum and breast milk IgG and IgA antibodies to ancestral SARS-CoV-2 (Wuhan-hu-1) and multiple variants of concern. The primary series significantly boosted the neutralizing capacity of breast milk against ancestral SARS-CoV-2, but not against Omicron BA.4/5. Future studies are needed to determine the extent to which maternal vaccination, including the variant-specific boosters, effectively protects breast-feeding infants against COVID-19.

## Figures and Tables

**Table 1:** Participant characteristics at enrollment or first vaccine dose.

Characteristics	Mean (SD) [Range] or *n* (%)
Age, years	33 (4) [27–42]
Race American Indian, Asian, or Black White Other	1/11 (9.1%) 9/11 (81.8%) 1/11 (9.1%) ^a^
Ethnicity Non-Hispanic Hispanic	11/11 (100%) 0/100 (0%)
Pregnant at time of first dose	3/11 (27.3%)
Gestational age at delivery, weeks	38.3 (2.86) [30–40]
Fetus/Infant age at first dose, weeks Fetus (n = 3) Infant (n = 8)	33 (3.6) [29–36] 6.8 (4.4) [1.9–13.1]
Vaccine manufacturer Moderna (mRNA-1273) Pfizer-BioNTech (BNT162b2)	4/11 (36.4%) 7/11 (63.6%)

^a^ Identified as white and black.

## Data Availability

The data presented in this study are available on request from the corresponding author.

## Supplementary Materials

The following supporting information can be downloaded at: https://www.mdpi.com/article/10.3390/vaccines11111643/s1, Table S1: Timing of sample collection relative to most recent vaccine dose.

## References

[1] Watson O.J., Barnsley G., Toor J., Hogan A.B., Winskill P., Ghani A.C. (2022). Global impact of the first year of COVID-19 vaccination: A mathematical modelling study. Lancet Infect. Dis..

[2] Children and COVID-19 Vaccination Trends: Summary of Data Publicly Reported by the Centers for Disease Control and Prevention. https://www.aap.org/en/pages/2019-novel-coronavirus-covid-19-infections/children-and-covid-19-vaccination-trends/.

[3] COVID-19 Vaccinations in the United States. https://covid.cdc.gov/covid-data-tracker/#vaccinations_vacc-people-booster-percent-pop5.

[4] Hospitalization of Infants and Children Aged 0–4 Years with Laboratory-Confirmed COVID-19—COVID-NET, 14 States, March 2020–February 2022. https://www.cdc.gov/mmwr/volumes/71/wr/mm7111e2.htm.

[5] Hunagund S., Golan Y., Asiodu I.V., Prahl M., Gaw S.L. (2022). Effects of Vaccination Against Influenza, Pertussis, and COVID-19 on Human Milk Antibodies: Current Evidence and Implications for Health Equity. Front. Immunol..

[6] Perl S.H., Uzan-Yulzari A., Klainer H., Asiskovich L., Youngster M., Rinott E., Youngster I. (2021). SARS-CoV-2-Specific Antibodies in Breast Milk After COVID-19 Vaccination of Breastfeeding Women. JAMA.

[7] Scrimin F., Campisciano G., Comar M., Ragazzon C., Davanzo R., Quadrifoglio M., Giangreco M., Stabile G., Ricci G. (2022). IgG and IgA Antibodies Post SARS-CoV-2 Vaccine in the Breast Milk and Sera of Breastfeeding Women. Vaccines.

[8] Rostad C.A., Chen X., Sun H.Y., Hussaini L., Lu A., Perez M.A., Hsiao H.M., Anderson L.J., Anderson E.J. (2022). Functional Antibody Responses to Severe Acute Respiratory Syndrome Coronavirus 2 Variants in Children with Coronavirus Disease 2019, Multisystem Inflammatory Syndrome in Children, and After Two Doses of BNT162b2 Vaccination. J. Infect. Dis..

[9] Chen X., Rostad C.A., Anderson L.J., Sun H.Y., Lapp S.A., Stephens K., Hussaini L., Gibson T., Rouphael N., Anderson E.J. (2021). The development and kinetics of functional antibody-dependent cell-mediated cytotoxicity (ADCC) to SARS-CoV-2 spike protein. Virology.

[10] Olearo F., Radmanesh L.S., Felber N., von Possel R., Emmerich P., Pekarek N., Pfefferle S., Norz D., Hansen G., Diemert A. (2022). Anti-SARS-CoV-2 antibodies in breast milk during lactation after infection or vaccination: A cohort study. J. Reprod Immunol..

[11] Pace R.M., Williams J.E., Jarvinen K.M., Belfort M.B., Pace C.D.W., Lackey K.A., Gogel A.C., Nguyen-Contant P., Kanagaiah P., Fitzgerald T. (2021). Characterization of SARS-CoV-2 RNA, Antibodies, and Neutralizing Capacity in Milk Produced by Women with COVID-19. mBio.

[12] Young B.E., Seppo A.E., Diaz N., Rosen-Carole C., Nowak-Wegrzyn A., Cruz Vasquez J.M., Ferri-Huerta R., Nguyen-Contant P., Fitzgerald T., Sangster M.Y. (2022). Association of Human Milk Antibody Induction, Persistence, and Neutralizing Capacity With SARS-CoV-2 Infection vs. mRNA Vaccination. JAMA Pediatr..

[13] Charepe N., Goncalves J., Juliano A.M., Lopes D.G., Canhao H., Soares H., Serrano E.F. (2021). COVID-19 mRNA vaccine and antibody response in lactating women: A prospective cohort study. BMC Pregnancy Childbirth.

[14] Collier A.Y., McMahan K., Yu J., Tostanoski L.H., Aguayo R., Ansel J., Chandrashekar A., Patel S., Apraku Bondzie E., Sellers D. (2021). Immunogenicity of COVID-19 mRNA Vaccines in Pregnant and Lactating Women. JAMA.

[15] Guida M., Terracciano D., Cennamo M., Aiello F., La Civita E., Esposito G., Gargiulo V., Maruotti G.M., Portella G., Sarno L. (2021). COVID-19 Vaccine mRNABNT162b2 Elicits Human Antibody Response in Milk of Breastfeeding Women. Vaccines.

[16] Jakuszko K., Koscielska-Kasprzak K., Zabinska M., Bartoszek D., Poznanski P., Rukasz D., Klak R., Krolak-Olejnik B., Krajewska M. (2021). Immune Response to Vaccination against COVID-19 in Breastfeeding Health Workers. Vaccines.

[17] Juncker H.G., Mulleners S.J., Coenen E.R.M., van Goudoever J.B., van Gils M.J., van Keulen B.J. (2022). Comparing Human Milk Antibody Response After 4 Different Vaccines for COVID-19. JAMA Pediatr..

[18] Juncker H.G., Mulleners S.J., van Gils M.J., de Groot C.J.M., Pajkrt D., Korosi A., van Goudoever J.B., van Keulen B.J. (2021). The Levels of SARS-CoV-2 Specific Antibodies in Human Milk Following Vaccination. J. Hum. Lact..

[19] Narayanaswamy V., Pentecost B.T., Schoen C.N., Alfandari D., Schneider S.S., Baker R., Arcaro K.F. (2022). Neutralizing Antibodies and Cytokines in Breast Milk After Coronavirus Disease 2019 (COVID-19) mRNA Vaccination. Obstet. Gynecol..

[20] Romero Ramírez D.S., Lara Pérez M.M., Carretero Pérez M., Suárez Hernández M.I., Martín Pulido S., Pera Villacampa L., Fernández Vilar A.M., Rivero Falero M., González Carretero P., Reyes Millán B. (2021). SARS-CoV-2 Antibodies in Breast Milk After Vaccination. Pediatrics.

[21] Yeo K.T., Chia W.N., Tan C.W., Ong C., Yeo J.G., Zhang J., Poh S.L., Lim A.J.M., Sim K.H.Z., Sutamam N. (2021). Neutralizing Activity and SARS-CoV-2 Vaccine mRNA Persistence in Serum and Breastmilk After BNT162b2 Vaccination in Lactating Women. Front. Immunol..

[22] Golan Y., Ilala M., Li L., Gay C., Hunagund S., Lin C.Y., Cassidy A.G., Jigmeddagva U., Matsui Y., Ozarslan N. (2023). Milk antibody response after 3(rd) COVID-19 vaccine and SARS-CoV-2 infection and implications for infant protection. iScience.

[23] Szczygioł P., Łukianowski B., Kościelska-Kasprzak K., Jakuszko K., Bartoszek D., Krajewska M., Królak-Olejnik B. (2022). Antibodies in the breastmilk of COVID-19 recovered women. BMC Pregnancy Childbirth.

[24] Selma-Royo M., Bauerl C., Mena-Tudela D., Aguilar-Camprubi L., Perez-Cano F.J., Parra-Llorca A., Lerin C., Martinez-Costa C., Collado M.C. (2022). Anti-SARS-CoV-2 IgA and IgG in human milk after vaccination is dependent on vaccine type and previous SARS-CoV-2 exposure: A longitudinal study. Genome Med..

[25] Perez S.E., Luna Centeno L.D., Cheng W.A., Marentes Ruiz C.J., Lee Y., Congrave-Wilson Z., Powell R.L., Stellwagen L., Pannaraj P.S. (2022). Human Milk SARS-CoV-2 Antibodies up to 6 Months After Vaccination. Pediatrics.

[26] Bauerl C., Zulaica J., Rusu L., Moreno A.R., Perez-Cano F.J., Lerin C., Mena-Tudela D., Aguilar-Camprubi L., Parra-Llorca A., Martinez-Costa C. (2023). Assessment of SARS-CoV-2 neutralizing antibody titers in breastmilk from convalescent and vaccinated mothers. iScience.

[27] Cabanillas-Bernal O., Cervantes-Luevano K., Flores-Acosta G.I., Bernaldez-Sarabia J., Licea-Navarro A.F. (2022). COVID-19 Neutralizing Antibodies in Breast Milk of Mothers Vaccinated with Three Different Vaccines in Mexico. Vaccines.

[28] Low J.M., Gu Y., Ng M.S.F., Wang L.W., Amin Z., Zhong Y., MacAry P.A. (2022). Human Milk Antibodies after BNT162b2 Vaccination Exhibit Reduced Binding against SARS-CoV-2 Variants of Concern. Vaccines.

[29] Chatterjee S., Bhattacharya M., Nag S., Dhama K., Chakraborty C. (2023). A Detailed Overview of SARS-CoV-2 Omicron: Its Sub-Variants, Mutations and Pathophysiology, Clinical Characteristics, Immunological Landscape, Immune Escape, and Therapies. Viruses.

[30] Schwartz A., Nir O., Toussia-Cohen S., Leibovich L., Strauss T., Asraf K., Doolman R., Sharabi S., Cohen C., Levin E.G. (2021). Presence of SARS-CoV-2 antibodies in lactating women and their infants following BNT162b2 messenger RNA vaccine. Am. J. Obstet. Gynecol..

[31] Breast Feeding Report Card. https://www.cdc.gov/breastfeeding/data/reportcard.htm#:~:text=At%206%20months%2C%2055.8%25%20of,breast%20milk%20at%206%20months.

[32] Regan A.K., Kaur R., Nosek M., Swathi P.A., Gu N.Y. (2022). COVID-19 vaccine acceptance and coverage among pregnant persons in the United States. Prev. Med. Rep..

[33] Villar J., Ariff S., Gunier R.B., Thiruvengadam R., Rauch S., Kholin A., Roggero P., Prefumo F., do Vale M.S., Cardona-Perez J.A. (2021). Maternal and Neonatal Morbidity and Mortality Among Pregnant Women with and without COVID-19 Infection: The INTERCOVID Multinational Cohort Study. JAMA Pediatr..

