# Supplementary material for: SARS-CoV-2 Antibody Profiles in Maternal Serum and Breast Milk Following mRNA COVID-19 Vaccination: A Longitudinal Prospective Observational Cohort Study
Source: Vaccines (Basel). 2023 Oct 26;11(11):1643. doi: 10.3390/vaccines11111643 (PMC10675665; doi:10.3390/vaccines11111643)
Supplement: Supplementary file 1 [file vaccines-11-01643-s001.zip › vaccines-2630437-supplementary.pdf]

Supplementary Data

Supplemenatry Table S1. Timing of sample collection relative to most recent vaccine dose.

Time Point	Range of Days*	Weeks Included*	Median Days*	Number of Samples Included
**Pre-Dose**	-14-0	-2 to 0	-2	8
**1-2 weeks Post 1**	7-14	1 to 2	13.5	4
**3-4 weeks Post 1**	18-28	3 to 4	21	9
**Post 2**	7-18	1 to 3	14	9
**1-month**	26-41	4 to 7	28	10
**2-months**	56-71	8 to 11	62	6
**3-months**	79-107	12 to 16	95	9
**4-6 months**	119-201	17 to 30	161	10
**7-9 months**	215-259	31 to 37	259	5

*Timing is relative to most recent vaccine dose.
